# Polymer/Fullerene Blend Solar Cells with Cadmium Sulfide Thin Film as an Alternative Hole-Blocking Layer

**DOI:** 10.3390/polym11030460

**Published:** 2019-03-11

**Authors:** Murugathas Thanihaichelvan, Selvadurai Loheeswaran, Kailasapathy Balashangar, Dhayalan Velauthapillai, Punniamoorthy Ravirajan

**Affiliations:** 1 Department of Physics, University of Jaffna, Jaffna 40000, Sri Lanka; thanihai@gmail.com; 2 Department of Physical Science, Trincomalee campus, Eastern University, Trincomalee 31010, Sri Lanka; loheeswaran@yahoo.com (S.L.); k_balashangar@yahoo.com (K.B.); 3 Faculty of Engineering and Science, Western Norway University of Applied Sciences, P.O. Box 7030, 5020 Bergen, Norway; Dhayalan.Velauthapillai@hvl.no

**Keywords:** CdS, P3HT, PCBM, bulk heterojunction, polymer blend solar cells, hole-blocking layer, chemical bath deposition

## Abstract

In this work, chemical bath-deposited cadmium sulfide (CdS) thin films were employed as an alternative hole-blocking layer for inverted poly(3-hexylthiophene) (P3HT) and phenyl-C61-butyric acid methyl ester (PCBM) bulk heterojunction solar cells. CdS films were deposited by chemical bath deposition and their thicknesses were successfully controlled by tailoring the deposition time. The influence of the CdS layer thickness on the performance of P3HT:PCBM solar cells was systematically studied. The short circuit current densities and power conversion efficiencies of P3HT:PCBM solar cells strongly increased until the thickness of the CdS layer was increased to ~70 nm. This was attributed to the suppression of the interfacial charge recombination by the CdS layer, which is consistent with the lower dark current found with the increased CdS layer thickness. A further increase of the CdS layer thickness resulted in a lower short circuit current density due to strong absorption of the CdS layer as evidenced by UV-Vis optical studies. Both the fill factor and open circuit voltage of the solar cells with a CdS layer thickness less than ~50 nm were comparatively lower, and this could be attributed to the effect of pin holes in the CdS film, which reduces the series resistance and increases the charge recombination. Under AM 1.5 illumination (100 mW/cm^2^) conditions, the optimized PCBM:P3HT solar cells with a chemical bath deposited a CdS layer of thickness 70 nm and showed 50% power conversion efficiency enhancement, in comparison with similar solar cells with optimized dense TiO_2_ of 50 nm thickness prepared by spray pyrolysis.

## 1. Introduction

Organic bulk heterojunction solar cells have attracted significant attention due to their potential to fabricate flexible and cost efficient solar cells [1]. Recent works reported that the efficiency has gone beyond the 10% mark with non-fullerene acceptors and high hole mobility polymers with multiple layers of donor acceptor composites [2,3,4]. The bulk heterojunction (BHJ) blend structure resolves the limitations of polymers, such as low diffusion lengths and low mobility, by providing interpenetrating donor–acceptor networks [5,6,7]. The most general structures used in high efficiency BHJ solar cells are transparent conducting oxide (TCO), electron-blocking layer (EBL), donor–acceptor blend/hole-blocking layer (HBL), and metal electrodes. Indium tin oxide (ITO) and fluorine-doped tin oxide (FTO) are the most commonly used TCOs and poly(3,4-ethylenedioxythiophene) poly(styrenesulfonate) (PEDOT:PSS) is well known as a hole- blocking layer (HBL) in these types of cells. Anyhow, the poor stability of PEDOT: PSS in an ambient atmosphere makes the conventional cell structure with PEDOT:PSS as HBL unstable [8].

The inverted structure consisting of TCO, HBL, acceptor-donor blend, EBL, or low work function metal electrodes (for hole collection), creates relatively stable BHJ solar cells [9]. Efficient charge collection at the electrodes of inverted bulk heterojunction solar cells is crucial for ensuring high efficiency [10]. Many inorganic materials—especially metal oxides such as TiO_2_ [11,12], SnO_2_ and ZnO [13,14]—were successfully used as the HBL in inverted BHJ solar cells due to the high electron mobility and optical transparency of these semiconductor materials.

CdS is an n-type semiconducting material with a direct bandgap of 1.42 eV, which is widely studied as a window material in CdTe and CuInGaSe thin film solar cells [15]. Relatively higher electron mobility makes CdS a suitable material for HBL in bulk heterojunction solar cells [16,17,18,19]. CdS thin films can be fabricated on the nanoscale using several methods including SILAR [20], atomic layer deposition [16], chemical vapor deposition [21], close space sublimation [22], and chemical bath deposition (CBD) [15,23]. Of all these, CBD is a simple and efficient method to fabricate CdS thin film using the solution process method at low temperatures. Despite higher electron mobility, CdS has strong UV absorption with an absorption edge of 520 nm. Hence, the CdS film thickness is crucial in determining the photovoltaic performance of the factor of CdS/PCBM:P3HT solar cells.

In this work, we focused on a simple chemical bath deposition method for fabricating the CdS thin film with different film thicknesses, and study the effect of the film thickness on the photovoltaic performance of bulk heterojunction fullerene/polymer blend solar cells. We chose P3HT and PCBM as the donor–acceptor pair as they are available commercially. 

## 2. Materials and Methods

### 2.1. CdS Thin Film Fabrication

CdS thin films were grown on a cleaned ITO coated glass substrate using a simple CBD method. The CBD method adopted was reported in detail elsewhere [15,24,25]. Aqueous solutions of 33 mM cadmium chloride (CdCl_2_) (99.99%, Sigma Aldrich), 66 mM thiourea ((NH_2_)_2_CN) (99%, Sigma Aldrich), 1 M ammonium chloride (NH_4_Cl) (99.9%, Sigma Aldrich), and 1 M ammonium hydroxide (NH_4_OH) (Merck) were used as precursors. The reaction chamber was filled with 550 mL of deionized water and heated to 80 °C. Then, using ultra-sonication, the heated water was degassed. Then the reaction chamber was heated to 85 °C, and stirred at a constant rate of 240 rpm using a magnetic stirrer. The cleaned ITO-coated glass substrates were kept vertically in a way that the ITO coated surface faced the center of the reaction bath. The prepared solutions of 15 mL NH_4_OH, 7.5 mL CdCl_2_, and 4 mL NH_4_Cl were added at an interval of 1 min and the temperature of the bath was increased up to 93 °C. Then the thiourea solution was titrated by 2 mL doses four times at an interval of 1 min. The system was kept at a constant temperature of 93 °C until the samples were removed. CdS layers with different layer thicknesses were coated on the ITO-coated glass substrate by depositing CdS for 17, 27, 37, and 47 min after the last titration of thiourea solution. The ITO substrates with a deposited CdS layer were cleaned by ultra-sonication for 10 s followed by an ethanol wash to remove the loosely attached CdS particles on the surface. The optical absorption spectra of the CdS electrodes were obtained using a UV–Vis spectrometer (JENWAY-6800, Staffordshire, UK) and the film thicknesses were measured using a Dektak surface profilometer. 

### 2.2. TiO_2_ Thin Film Fabrication

TiO_2_ thin film on the ITO substrate was fabricated by simple spray pyrolysis as reported earlier [26,27]. The precursor solution for the spray was prepared as follows. Titanium isopropoxide (TIP) (97%, Sigma-Aldrich) was dispersed in Acetylacetone (AcAc) (Sigma-Aldrich) at a volume ratio 1:1.41. Then the TIP-AcAc suspension was diluted in absolute ethanol (99.9%) at a volume ratio of 1:9. The prepared solution was mixed for 30 min using a magnetic stirrer at a spin rate of 300 rpm. Then, 1 mL of precursor solution was fed into a spray gun (Badger Airbrush 100, USA) and sprayed using 99.9% nitrogen as the carrier gas. The ITO substrate that was to be sprayed was preheated to 450 °C on a hotplate and sprayed until the precursor solution was emptied. Sprayed TiO_2_ films on the ITO substrates were sintered at 450 °C for 30 min for further crystallization. 

### 2.3. Solar Cell Fabrication

**Figure 1:**
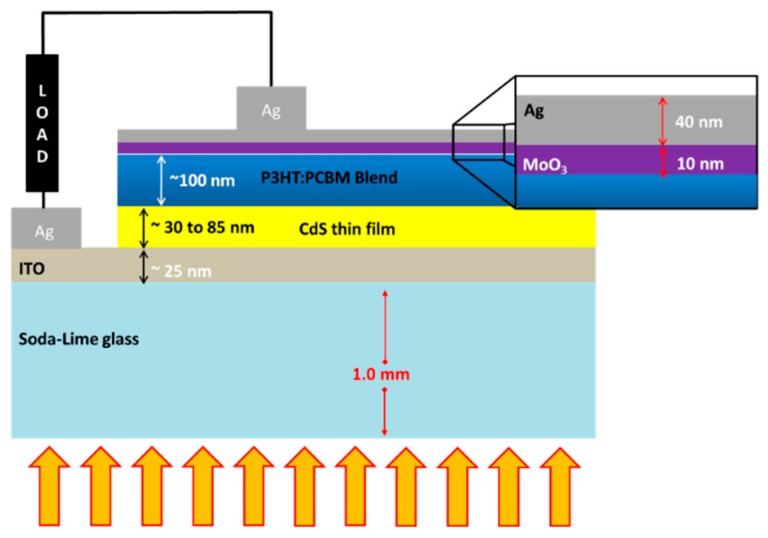
Schematic of the fabricated inverted device with an external load used for electrical measurements. The arrow marks indicate the shining of the light. (Not drawn to scale).

The CdS film-deposited ITO glass substrate was cleaned with acetone, isopropanol and dried in nitrogen. CdS films were first heated at 110 °C for 10 min in order to remove the surface moisture. A chlorobenzene (CB) solution of P3HT:PCBM (1:1 by weight) containing 25 mg/mL P3HT and 25 mg/mL PCBM was prepared by stirring in a nitrogen filled glove box at 60 °C and kept overnight. The solution was allowed to cool down to room temperature, and was then filtered with a 0.2 μm polytetrafluoroethylene filter. The as-prepared P3HT/PCBM blend solution was spin-coated at 1250 rmp for 30 s onto the ITO glass substrate coated with CdS thin films that were pretreated with 50 W oxygen plasma at 200 mTorr for 10 min. The samples were then kept at 140 °C for 1 min to allow the self-organization of P3HT, as well as to remove residual solvent and to some extent improve the contact between the polymer and CdS film. The top contact electrode was made by the evaporation of silver using a thermal evaporator (Edwards E306) after the deposition of a MoO_3_ layer under high vacuum (2 × 10^−6^ mTorr). Figure 1 schematically illustrates the device structure with an appropriate layer thicknesses. The electrical characterization of the solar cells was carried out using a computer interfaced source measure unit (Keithley 2601, Cleveland, OH, USA) under 100 mW/cm^2^ illumination using a solar simulator (SCIENCETECH, ON, Canada) with an AM 1.5 filter.

## 3. Results and Discussion

**Figure 2:**
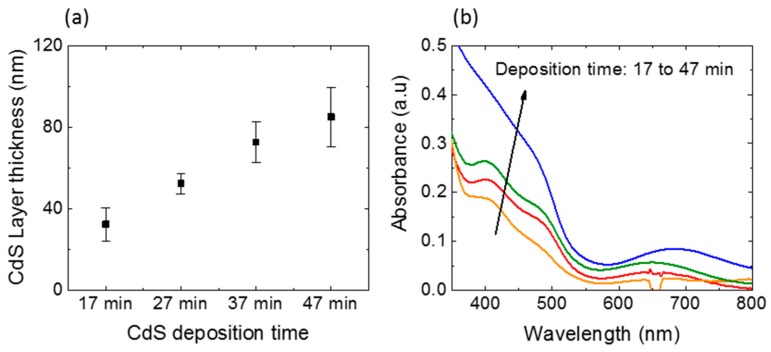
(**a**) Variation of CdS film thickness and (**b**) optical absorption spectra of CdS thin films fabricated with different deposition time.

The thickness variation of chemical bath-deposited CdS films with the deposition time is given in Figure 2a. The thicknesses were measured at five different points and the error bars represents the standard deviation values. A linear increase in average thickness was observed within the range of deposition time. This showed the uniform growth of CdS films within the deposition time of 47 min. The average thicknesses were measured as 32.3 ± 8 nm (~30 nm), 52.3 ± 5 nm (~50 nm), 72.67 ± 10 nm (~70 nm) and 86.0 ± 14.5 nm (~85 nm) for the deposition times of 17, 27, 37 and 47 min, respectively. This confirmed that the CdS layer thicknesses can be controlled at the nanometer scale by controlling the deposition time, as reported in [28]. The UV-Vis spectra of the fabricated CdS thin films are shown in Figure 2b. The fabricated films showed a strong absorption in the UV region with a sharp absorption edge at 520 nm, which represents the direct bandgap (2.42 eV) of the CdS. This is in good agreement with other works [28,29,30]. The sharp absorption edge also ensures the presence of highly crystalline CdS in the thin film. Moreover, the film was transparent in the visible region regardless of the deposition time, which is an essential criterium for a window material in solar cell applications. As expected, absorption by CdS thin films in the UV region increased with the increasing deposition time, which is consistent with the literature [15,28,31,32].

**Figure 3:**
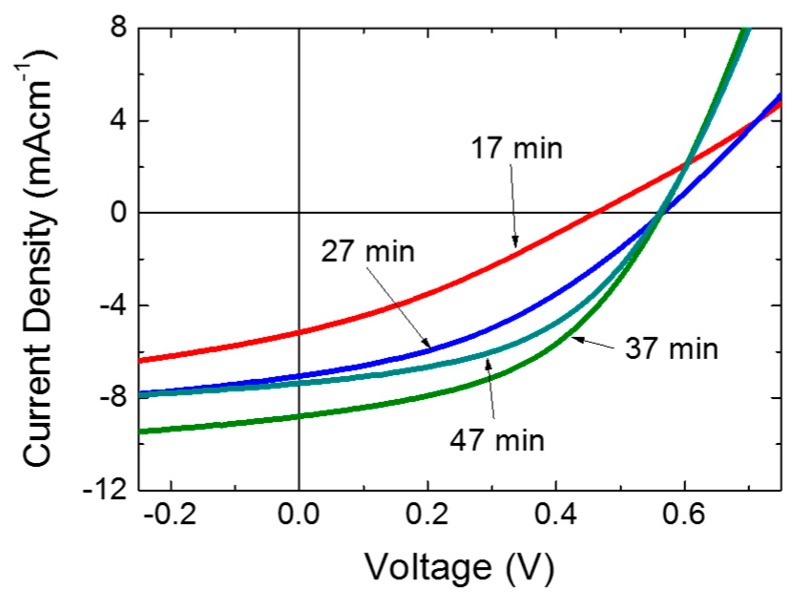
J-V curve ITO/CdS/P3HT:PCBM blend/PEDOT:PSS/MoO_3_/Ag devices with different CdS deposition times.

**Figure 4:**
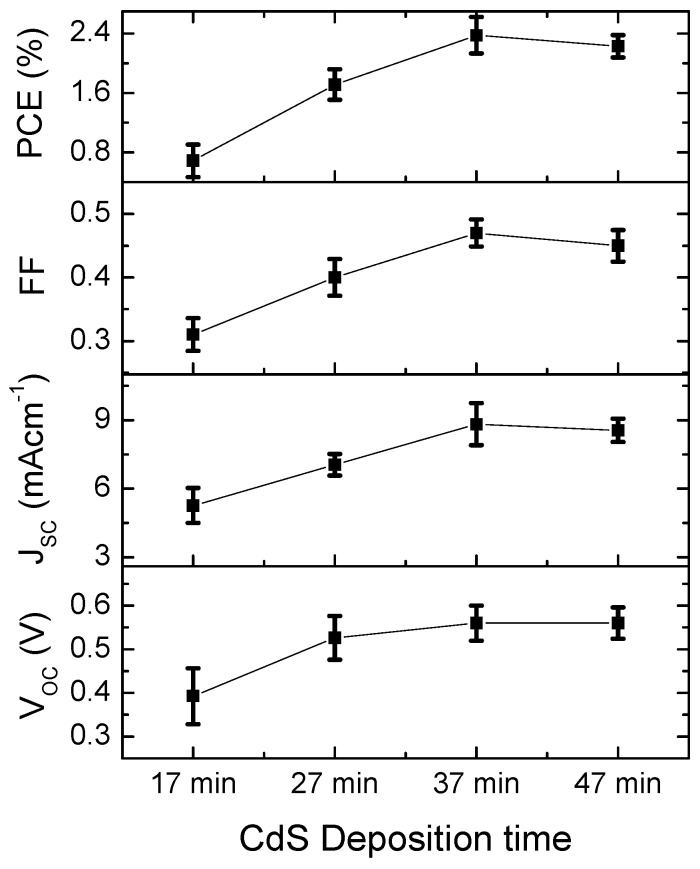
Variation of PV device properties of ITO/CdS/PCBM:P3HT blend/PEDOT:PSS/MoO_3_/Ag devices with different CdS deposition times.

The J-V characteristics of the solar cells fabricated with different CdS film thicknesses under simulated illumination of 100 mW/cm^2^ (one sun) are shown in Figure 3. The average figures of merits of four identical solar cells with different CdS deposition times (layer thicknesses) are summarized in Figure 4. As seen in Figure 4, the average V_OC_ of the solar cells increased from 0.39 to 0.57 when the CdS film thickness increased from ~30 to ~50 nm. The average V_OC_ remained unchanged with the further increment in deposition time. The lower V_OC_ of solar cells with ~30 nm CdS film can be attributed to the shunting of the active layer with the ITO due to the presence of pin holes in the thin CdS film with high rms roughness when compared to the film thickness [15]. The unchanged V_OC_ at higher CdS film thicknesses (~50 nm to ~85 nm) ensures that the CdS film is pin hole free at higher deposition times [15]. The variation of J_SC_ and fill factor showed similar trends with the increasing CdS deposition time. Both increased up to ~70 nm and a slight reduction was observed in devices with ~85 nm CdS film. The increment in J_SC_ and fill factor can be explained by the effective isolation provided by the thick CdS film. At optimum thickness, the electron extraction and transport to the ITO layer is at its best and yields a better J_SC_ and fill factor. The slight drop in the J_SC_ and fill factor of devices with an ~85 nm CdS film can be explained by the strong UV absorption with an increased CdS layer thickness. Overall, the power conversion efficiency replicates the trend in the J_SC_ and fill factor, and increased with the CdS film thickness up to ~70 nm and decreased with thicker CdS layers. The optimum PCE of 2.8% was obtained from the devices with a ~70 nm thick CdS film, which is in the similar range reported for BHJ solar cells with CdS films fabricated by employing different physical vapor deposition methods (see Table 1). 

As summarized in Table 1, we emphasize that the J_SC_ and V_OC_ of our champion device are the highest among all the reported works on CdS/PCBM:P3HT blend-based devices that we are aware of. However, the PCE of our device was not the highest because of the low fill factor. The low fill factor can be correlated to the roughness of the CdS film. The surface roughness of the chemical bath-deposited CdS films was higher when compared to other deposition methods.It has also been reported that the roughness of the CdS film fabricated by CBD decreases with increasing deposition time [15,33,34,35]. Achieving the highest J_SC_ with our device compared to the devices in the table reiterates that optimizing HBL thickness is essential. It is worth noting that the maximum V_OC_ of all the reported works was in the range of 0.60 to 0.62 V. This confirms that the V_OC_ is determined by the energy difference between the HOMO of the polymer donor (P3HT) and the LUMO level of the acceptor (PC_61_BM).

**Figure 5:**
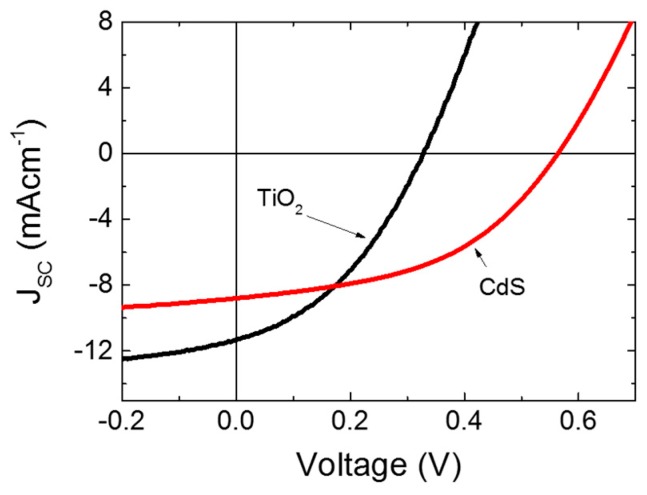
The J-V measurements of P3HT:PCBM solar cells with TiO_2_ and CdS thin film as HBL with respective optimum thicknesses.

**Figure 6:**
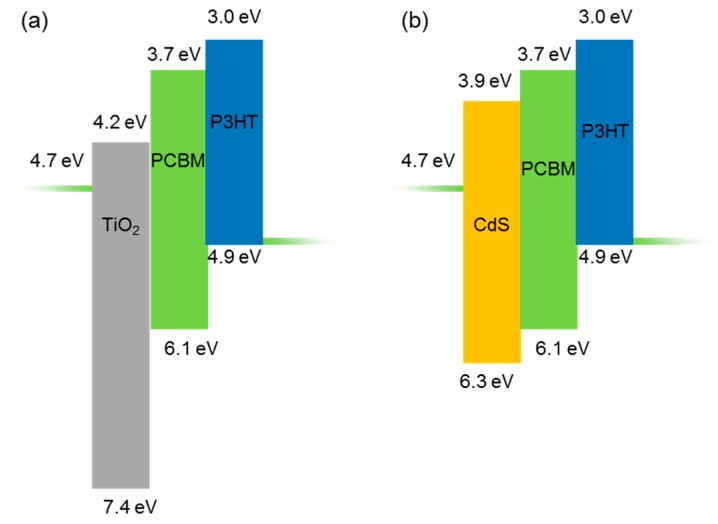
Proposed electronic energy level alignment of a P3HT:PCBM BHJ solar cells with (**a**) TiO_2_ and (**b**) CdS as the hole-blocking layer with ITO and MoO_3_/Ag as bottom and top electrodes, respectively.

Finally, we compared the performance of the PCBM/P3HT blend with an optimized CdS layer with a similar device that had a TiO_2_ thin film of the same thickness fabricated by spray pyrolysis as the hole-blocking layer. Figure 5 shows the J-V curves of the devices with TiO_2_ and CdS as hole-blocking layers. The TiO_2_ devices showed a higher J_SC_ of 11.1 mA cm^−1^ against the 9.04 mA cm^−1^ of the CdS device. The higher J_SC_ of the TiO_2_ devices can be attributed to the lower filtering effect of TiO_2_ due to its higher bandgap. However, the CdS device showed a higher average V_OC_ of 0.59 V against 0.33 V of TiO_2_ devices. Our CdS device had a higher PCE (2.81%) when compared to the devices with TiO_2_ as the hole-blocking layer (2.0%). The improved V_OC_ of the CdS devices can be explained by the appropriate band diagrams of the solar cells [25,36]. As reported earlier, the V_OC_ of polymer-based solar cells are limited by the difference between the HOMO of the donor and the LUMO of the acceptor [37,38]. Figure 6a,b compares the electronic energy levels of the solar cells with TiO_2_ and CdS hole-blocking layers, respectively. The values mentioned in the figure are below the ground level. The higher conduction band edge of the CdS layer in comparison with the conduction band edge of TiO_2_ explains the higher V_OC_ found in CdS/PCBM:P3HT solar cells [31]. 

## 4. Conclusions

In conclusion, the influence of the CdS layer thickness on the performance of CdS/PCBM:P3HT solar cells was systematically studied by successfully growing the CdS films using a simple chemical bath deposition method. PCBM/P3HT solar cells made with CdS films showed a strong effect of the CdS layer thickness on the power conversion efficiency of CdS/PCBM:P3HT solar cells. The CdS layer fabricated with CdS layer that was 70 nm thick showed optimized device performance with an average power conversion efficiency of 2.4%, and a champion cell showed a power conversion efficiency of over 3.0% under the illumination of 100 mW/cm^2^ at AM 1.5 conditions. We also compared the optimum device with TiO_2_ as the hole-blocking layer. The result suggested that the CdS with tailored thickness can be used as an effective hole-blocking layer in bulk heterojunction solar cells with higher open circuit voltages.

## Figures and Tables

**Table 1:** Comparison of P3HT:PCBM BHJ solar cells with the CdS hole-blocking layer fabricated by different techniques.

CdS Thin Film Fabrication Method	Optimum CdS Layer Thickness	Optimum Device Performance				Ref.
		J_SC_ *	V_OC_^#^	FF	PCE (%)	
Thermal decomposition of cadmium xanthate precursor	10 nm	8.28	0.60	0.63	3.22	[19]
Spraying equimolar solutions of CdCl_2_ and CS(NH_2_)_2_	330 nm	1.91	0.502	0.30	0.29	[17]
Chemical bath deposition	21.3 nm	5.33	0.62	0.43	1.42	[18]
Atomic layer deposition	17.5 nm	8.94	0.61	0.61	3.33	[16]
Chemical bath deposition	~70 nm	9.04	0.59	0.48	2.81	This work

* Short circuit current density in mA cm^−1^, ^#^ Open circuit voltage in V.
